# Medicine shortages: a commentary on causes and mitigation strategies

**DOI:** 10.1186/s12916-016-0674-7

**Published:** 2016-09-29

**Authors:** Swathi Iyengar, Lisa Hedman, Gilles Forte, Suzanne Hill

**Affiliations:** World Health Organization, 20 27 Avenue Appia, Geneva, CH-1211 Switzerland

**Keywords:** Shortage, Essential medicines, Ethics, Supply chain management

## Abstract

Shortages of medicines and vaccines have been reported in countries of all income levels in recent years. Shortages can result from one or multiple causes, including shortages of raw materials, manufacturing capacity problems, industry consolidation, marketing practices, and procurement and supply chain management. Existing approaches to mitigate shortages include advance notice systems managed through medicine regulatory authorities, special programmes that track medicines, and interventions to improve efficiency of the medicine supply chain. Redistribution of supplies at the national level can mitigate some shortages in the short term. International redistribution and exceptional regulatory approvals may be used in limited circumstances, with the understanding that such approaches are complex and may introduce cost and quality risks. If it is necessary to prioritise patients to receive a medicine that is in shortage, evidence-based practice should be used to ensure optimal allocation. Important steps in reducing medicine shortages and their impact include identifying medicines that are most at risk, developing reporting systems to share information on current and emerging shortages, and improving data from medicine supply chains.

## Background

Shortages of medicines and vaccines have been reported in countries of all income levels in recent years. For example, in 2013, Kerala, India, faced a shortage of 130 medicines on the state’s Essential Medicines List and Rationalized Drug List when suppliers did not respond to state tenders [1]. Chemotherapeutic agents, generic injectables, and epilepsy medications continue to be in short supply across North America for reasons ranging from manufacturing problems to low commercial interest [2, 3]. The BCG vaccine, critical in childhood immunization and also in the treatment of bladder cancer, has been in shortage across multiple markets since 2012 due to manufacturing quality problems combined with high demand [4].

Medicine shortages result from single or multiple causes, ranging from problems at the production level to weak supply chains that prevent medicines from reaching points of care. Irrespective of the causes, shortages lead to increased costs for health systems. The additional labour cost of responding to shortages in the United States was estimated at US$ 216 million per annum by Kaakeh et al. [5]. Other costs to health systems include higher prices of substitute medicines, as well as costs of treating adverse reactions, medication errors, and consequences of delayed therapy [6]. Shortages may also lead to prescribers and dispensers substituting medicines that are not clinically appropriate.

In this article, we outline the causes of shortages and the existing solutions to mitigate these, as well as the ethical considerations and the actions taken by the global community to manage shortages.

## Reasons for shortages

Shortages result from one or more causes [7], including manufacturing issues, acute healthcare needs, external political and economic factors, or marketing, procurement, and supply chain management practices. Examples of manufacturing issues resulting in shortages include a lack of raw materials, limited manufacturing capacity, or product quality problems resulting in more stringent inspections and plant closures. Health emergencies, such as disasters and disease outbreaks, can also trigger shortages due to unexpected and large surges in demand [7]. Similarly, changes in recommended clinical practices can dramatically impact availability; for example, when the United Kingdom recommended inclusion of the meningitis B vaccine in the childhood immunization programme in 2015, a surge in demand led to a shortage of supply [8].

Industry consolidation has led to fewer manufacturers for older, often less profitable medicines [9]. Consolidations may also lead to new regulatory applications and transfers in marketing authorization, with resulting requirements for regulatory assessment [10]. Incomplete or delayed regulatory submissions may delay marketing authorization and potentially lead to national supply shortages. Factors such as poor financial incentives for manufacturers, changes in reimbursement, changes in regulatory requirements, and costs required to correct manufacturing errors can decrease the availability of medicines [9]. If profit margins are low, for example due to either competition or price controls, the expenses required to ensure good manufacturing practices [11] may not be covered and, therefore, keeping production lines in operation may not be viable [12].

At present, there is no global system to capture data or trends on shortages, which compromises efforts to respond to or prevent them; some national early warning systems do exist, but only from a limited number of countries and programs. Additionally, the criteria for what constitutes a medicine at high risk of shortage may differ from region to region.

## Management of shortages

Efforts to avoid and mitigate shortages include advance notice systems to national medicines regulatory authorities (NMRAs), as well as special initiatives that monitor stock levels of specific medicines. Data from medicine supply chains are important for maintaining and forecasting supply. Advance notice systems with NMRAs exist mainly in high-income markets and require manufacturers to advise them regarding impending shortages [13]. Responses from NMRAs include identifying clinically acceptable substitute medicines, providing notifications to prescribers and dispensers, facilitating exceptional market authorizations for other producers and, in some cases, identifying other manufacturing capacities; however, NMRAs do not have legislative authority to compel a manufacturer to produce.

Professional associations, such as the American Society of Health-System Pharmacists (ASHSP) and the Association Africaine des Centrales d’Achats de Médicaments Essentiels (ACAME), an association of central medical stores across Africa, have mechanisms for sharing information among members. The ASHSP collates and reports national wholesale level shortages and publishes these online [14]. Similarly, ACAME shares information on pricing, procurement sources, availability, quality, and technical specifications of medicines. One of its aims is to coordinate demand across countries as a means of reducing shortages at wholesale and supply chain levels in the region [15]. These types of systems are also useful in managing shortages, for example, by identifying alternative sources of products. However, this information comes primarily from procurement and logistics management information systems, which may not immediately signal that a shortage is caused by a manufacturing problem.

Data from the medicines supply chain are useful, particularly for analysing trends and maintaining appropriate supply levels to prevent avoidable shortages [16]. Still, challenges with unavailable or unreliable supply chain data have persisted for decades [17]. Monitoring the supply chain may also allow countries to redistribute medicines across warehouses or health facilities, as well as to manage emergency orders when necessary. In theory, countries could work together to mitigate shortages by sharing or redistributing inventories; however, this option would require regulatory agreements and may introduce extra costs and quality risks [7]. Illegal diversion of stock may create or exacerbate shortages in the originating country, distort overall demand, and may result in the introduction of substandard, spurious, falsely labelled, falsified, or counterfeit (SSFFC) products into the supply chain [18, 19].

Ethical approaches to managing medicines in short supply can be complicated, as seen, for example, in the inactivated polio vaccine and cancer therapies shortages across multiple countries [20, 21]. An ethical framework to ensure equity, fairness and public health interest is therefore important in managing a shortage [22]. For example, the allocation of medicines may be guided by evidence that prioritizes high-risk groups [23] or, in other cases, to groups that would have greater therapeutic benefit.

## Future directions and conclusions

Addressing medicine and vaccine shortages is a multifaceted issue. Ensuring access to essential medicines and health technologies is a target of the Sustainable Development Goals [24]. In addition to improving the quality and scope of a country’s national supply chain data, international collaboration is required in order to manage large-scale shortages. Further, definitions of shortages and stock-outs must be harmonized to develop effective cross-country interventions. Similarly, medicines most at risk of global shortages, especially those without clinically acceptable substitutes, must be identified and prioritized for global action. With an improved understanding of the gaps in current information on the causes of shortages and noting the benefits of global communication, countries may also consider supporting the development of a global early-warning shortage notification system to identify substitutes, alternative suppliers, or other mitigation measures. In the context of minimizing the risk of SSFFC, countries may consider implementing ‘track and trace’ systems for medicines. These types of mechanisms promote robust data collection and can strengthen good governance and accountability in the pharmaceutical sector. It is equally important that countries promote the optimal use of medicines to ensure accurate and clinically appropriate demand. Ultimately, policy mechanisms and harmonization efforts should emphasize their support for good manufacturing practices, appropriate supply chain management and security, and the coordination of strategies for the notification of shortages.
